# Enhanced tendon–bone healing with acidic fibroblast growth factor delivered in collagen in a rabbit anterior cruciate ligament reconstruction model

**DOI:** 10.1186/s13018-018-0984-x

**Published:** 2018-11-27

**Authors:** Daifeng Lu, Chuandong Yang, Zhitao Zhang, Mochao Xiao

**Affiliations:** 1 The Fourth Affiliated Hospital of Harbin Medical University, No. 37 Yiyuan street, Harbin, Nangang District China; 2 Heilongjiang Provincial Academy of Medical Sciences, No. 157 Care Road, Harbin, Nangang District China

**Keywords:** aFGF, Anterior cruciate ligament, Reconstruction, Tendon–bone healing

## Abstract

**Background:**

The objective of the present study was to investigate the effectiveness of acidic fibroblast growth factor delivered in collagen (aFGF/collagen) for promoting tendon–bone interface healing after anterior cruciate ligament (ACL) reconstruction in rabbits.

**Methods:**

ACL reconstructions were performed in the right hind limbs of New Zealand rabbits. Each left long digital extensor tendon was harvested as an autograft, and collagen incorporating different concentrations of aFGF or same amount of collagen alone was applied at the tendon–bone interface after ACL reconstruction. The control group underwent ACL reconstruction only. There were high and low aFGF/collagen groups, collagen alone group, and control group (*n* = 21 rabbits per group). Histological and biomechanical analyses were performed at 4, 8, and 12 weeks postoperatively to evaluate the effect of aFGF/collagen on tendon–bone interface healing.

**Results:**

Results of biomechanical tests showed that at both 8 and 12 weeks postoperatively, the elastic modulus and stiffness in both the high and low aFGF/collagen treatment groups were significantly higher than those in the control group and collagen alone group, with that in the high aFGF/collagen concentration group being the highest. Histological analysis showed that at 8 weeks, tightly organized Sharpey-like fibers were observed in both aFGF/collagen groups with new bone growth into the tendon in the high concentration group. At 12 weeks postoperatively, a fibrocartilage transition zone was observed in the bone tunnels in both aFGF/collagen groups, especially in the high aFGF/collagen group.

**Conclusion:**

Application of the aFGF/collagen composite could enhance early healing at the tendon–bone interface after ACL reconstruction, especially with the use of a high aFGF/collagen concentration.

## Background

In recent years, arthroscopic reconstruction of the anterior cruciate ligament (ACL) with the hamstring tendon as a graft has been widely used in clinical practice. The graft is biologically fixed for subsequent healing at the tendon–bone interface. This results in a long healing time and uncertain healing strength, which limit the early rehabilitation of patients and affect the final treatment outcome of ligament reconstruction. Thus, the healing at the tendon–bone interface has become an important indicator of successful ACL reconstruction [12].

Tendon–bone healing can be divided into indirect versus direct insertions [1]. Indirect insertions use collagen fibers to connect tendon grafts and bone tunnels. The direction of the fibers is usually perpendicular to the axial direction of the bone tunnel, and these are called the Sharpey-like fibers. Direct insertions are characterized by connection of the graft and bone tunnel through the fibrocartilage, similar to the normal fibrocartilage connection, forming the “tidal line” structure that stains positively for basophilias and can be found in the normal ACL tendon–bone end point or tendon–bone late healing phase at the exit of the bone tunnel [22]. Most researchers believe that indirect insertion is less effective than direct insertion, which can support restoration of mechanical strength by promoting complete healing between the transplanted tendon and the bone tunnel [21]. Thus, the promotion of early and effective tendon–bone healing during ACL reconstruction has become a hot topic [19].

At present, an autologous periosteal graft and platelet-rich plasma (PRP) are used to promote the healing of tendon and bone. The periosteum has strong osteogenic capacity, and it can also synthesize growth factors to induce cartilage formation [6]. However, the disadvantages of these donor tissues include the limited supply of donor periosteum, damage to the donor area, and dramatic differences in the activity and structure between two sides of the periosteum. PRP is an autologous enriched source of various growth factors. Although in vitro and animal studies have suggested that it promotes tendon–bone healing, its application in arthroscopic rotator cuff repair and ACL surgery failed to show a clear benefit [11].

In recent years, the application of biological growth factors to promote tendon–bone healing in the bone tunnel has attracted increasing attention [24], such as transforming growth factor (TGF), bone morphogenetic protein (BMP), basic fibroblast growth factor (bFGF), and granulocyte colony-stimulating factor (G-CSF) [17]. However, none of these growth factors was found to support formation of fibrocartilage tissue at the tendon–bone interface, partially because no carrier with a defined growth factor release speed has been found. Another member of the FGF family, the acidic FGF (aFGF, also known as FGF1) has a significant mitogenic effect on mesodermal-derived cells, including osteoblasts and chondrocytes, and can stimulate the formation of new capillaries [5]. In addition, aFGF can promote not only proliferation and maturation of chondrocytes, but also migration and colony formation during differentiation [3]. The application of aFGF may induce fibrocartilage formation at the tendon–bone interface, which also represents the formation of a direct contact. However, its effect on tendon–bone healing has yet to be investigated.

Collagen has been shown to have low antigenicity as well as good biocompatibility and biodegradability. It also plays an important role in cell proliferation, differentiation, and migration as well as in wound healing. Moreover, collagen has been employed previously as a drug release carrier [2]. Therefore, the incorporation of aFGF into collagen may potentially overcome the “synovial fluid infusion” effect. In addition, the aFGF concentration could possibly affect the local release of aFGF at the tendon–bone interface [23]. In this study, we investigated whether delivery of aFGF incorporated within collagen at the tendon–bone interface during ACL reconstruction could promote tendon–bone healing.

## Methods

### Study design

Eight-four healthy New Zealand rabbits (44 females and 40 males) with an average age of 6 months and an average weight of 4.5 kg were provided by the Experimental Animal Center of The Fourth Affiliated Hospital of Harbin Medical University. This double-blinded study was approved by the Institutional Animal Care and Use committee at the The Fourth Affiliated Hospital of Harbin Medical University and performed under the Guidelines for the Care and Use of Animals in Research. A statistical random number generator was used to generate a randomization list, and with stratification by gender, equal numbers of female and male rabbits were assigned to each group. This was a double-blinded study in that the individuals collecting, recording, and analyzing the data were blinded to group assignment, and data were stored by a third party until the experiments were completed.

The rabbits were randomly divided into four groups (*n* = 21/group) as follows: (1) ACL reconstruction with an autograft, and 4 μg aFGF incorporated into 15 ml collagen applied in the bone tunnel [20]; (2) ACL reconstruction with an autograft and or 1 μg FGF incorporated into 15 ml collagen in the bone tunnel; (3) ACL reconstruction with an autograft, and 15 ml collagen applied in the bone tunnel; (4) ACL reconstruction with an autograft only. Rabbits in each group were sacrificed at postoperative 4 (*n* = 7), 8 (*n* = 7), and 12 weeks (*n* = 7). For each group and time point, seven specimens were used for biomechanical analysis and then used for histological evaluation.

### Preparation of aFGF-incorporating collagen

The fat and fascia were removed from the fresh beef Achilles tendon, cleaned, and hardened in a − 20 °C refrigerator; the tendon was sliced into uniform pieces. About 100 g tendon was washed with deionized water and disinfected for 30 min using 75% alcohol. Then, the tendon was repeatedly washed using double distilled water, placed into 1 L 0.5 M glacial acetic acid, and kept at 4 °C for over 24 h with intermittent stirring. The expanded collagen was mixed in a high-speed tissue crusher with 4 L 0.5 M acetic acid. Then, 1 g pepsin was added and dissolved at 4 °C for more than 24 h. The supernatant was collected after centrifugation at 4000 r/min for 30 min at 4 °C. An equal volume of 20% NaCl solution was added to the supernatant for salting for over 48 h. The precipitated flocculent was dissolved in 2 L 0.5 M glacial acetic acid and filtered using the double-layer gauze. The resulting collagen solution was dialyzed with double distilled water for 5 days or more. The water was changed twice daily to produce the collagen solution. Finally, 1 μg or 4 μg aFGF (provided by Jinan University) was added into 15 ml of collagen solution for the two test treatments. After freeze-drying, aFGF/collagen complex with two concentrations of aFGF were obtained for the low-dose aFGF (0.067 μg/ ml) and high-dose aFGF groups (0.267 μg/ ml). All test samples were sterilized using ^60^Co.

### ACL reconstruction surgical protocol

The long digital extensor tendon was isolated from the left hind limb of the rabbit, and then, a medial parapatellar arthrotomy was made on the right hind limb to expose the ACL. The ACL was excised from the top and bottom end points. The bone tunnel was created using a 1.2-mm-diameter drill bit as the bottom end point. Similarly, bone tunnel was made on the femoral condyle bone as the upper end point. Both ends of the sutures attached to the graft were pulled out of the joint cavity and passed the ends of the graft through the bone tunnel. For fixation of the ends, a Kirschner wire was used to drill in the cortical bone slightly lower than the tibial tubercle, and one surgical thread was passed through the tunnel and tied into a knot with the end of another suture. Then, two single cortical bone tunnels were drilled on the femoral stem ligament end from outside to inside laterally using a Kirschner wire with a 0.3-cm interval. The two ends of the graft were passed through the bone tunnel with the knee bent at 90° and then secured with a tightened ligament knot.

Collagen was applied according to the following steps. One milliliter of collagen suspension and 0.5 ml thrombin were prepared in separate 1-ml syringes. Using a three-way valve, they were injected into the bone tunnel sequentially, and the incision was sutured layer-by-layer once the collagen had coagulated and attached within the bone tunnels at both ends of the graft. Ropivacaine was injected in the incision postoperatively, and rabbits were caged individually. Penicillin was injected intramuscularly for 3 days to prevent infection.

### Biomechanical analysis

At 4, 8, and 12 weeks postoperatively, seven animals from each group were sacrificed. The soft tissues surrounding the ACL and sutures were removed, and 5 cm residues of the femur and tibia were spared for fixation. The ACL graft was tested in a single arm-type electronic stretching machine (Instron 3342 Series Single Column System, Instron USA) and adjusted so that the axis of distraction was parallel to the long axis of the graft. The specimens were preconditioned with preload of 5 N; then, a load-to-failure test was performed with an elongation rate of 5 mm/min on the tibial tendon–bone interface. The machine recorded the load and displacement data and reported a load–displacement curve from which the ultimate stress value was retrieved. The stiffness and elastic modulus were calculated according to the curve. Saline solution spray was used to maintain specimen hydration throughout the testing procedure, and all tests were performed at room temperature.

### Histological analysis

At 4, 8, and 12 weeks postoperatively, specimens were harvested and the biomechanical analysis was carried out. Then, all specimens were fixed in 95 ml of 12% formaldehyde mixed with 5 ml glacial acetic acid at room temperature for 24 h. After fixation, the specimens were washed for 15 min and then decalcified using a 30% hydrochloric acid formaldehyde solution (30 ml hydrochloric acid + 10 ml formaldehyde + 60 ml water) for 24 h. The specimens were rinsed with water for 3 h and then rinsed with distilled water. The femoral tunnel and tibial tunnel were cut open along the longitudinal axis and trimmed to specimens 1 cm × 0.5 cm × 0.5 cm in size. The specimens were dehydrated in a gradient series of 75%, 85%, 95%, and 100% alcohol for 1.5 h, cleared twice in xylene for 1 h at room temperature, and embedded in paraffin heated to 60 °C. Five-micrometer-thick sections were stained with hematoxylin for 10 min and rinsed with water followed by hydrochloric acid alcohol and blue solution. Finally, the sections were co-stained with eosin for 20 s before observation of the tendon–bone interface under a microscope (BX43 Olympus Japan Olympus Co., Ltd.). The evaluation of histology was double-blinded to evaluators.

### Statistical analysis

All statistical analyses were performed using the SAS 9.3 statistical software package (SAS Institute Inc., Cary, NC, USA). The continuous variables are presented as mean ± standard deviation, and the categorical variables as frequency and percentage value. Categorical variables were compared using Fisher’s exact test. Repeated ANOVA followed by least significant difference (LSD) test was used to compare the means of continuous variables from different times and groups. *P* ≤ 0.05 was considered statistically significant.

## Results

All animals tolerated the ACL reconstruction surgical procedure well. At 4 days postoperatively, four rabbits had a minor infection at the incision that healed after treatment with antibiotics following debridement.

### Effect of FGF/collagen on ACL graft by histological evaluation

**Fig. 1:**
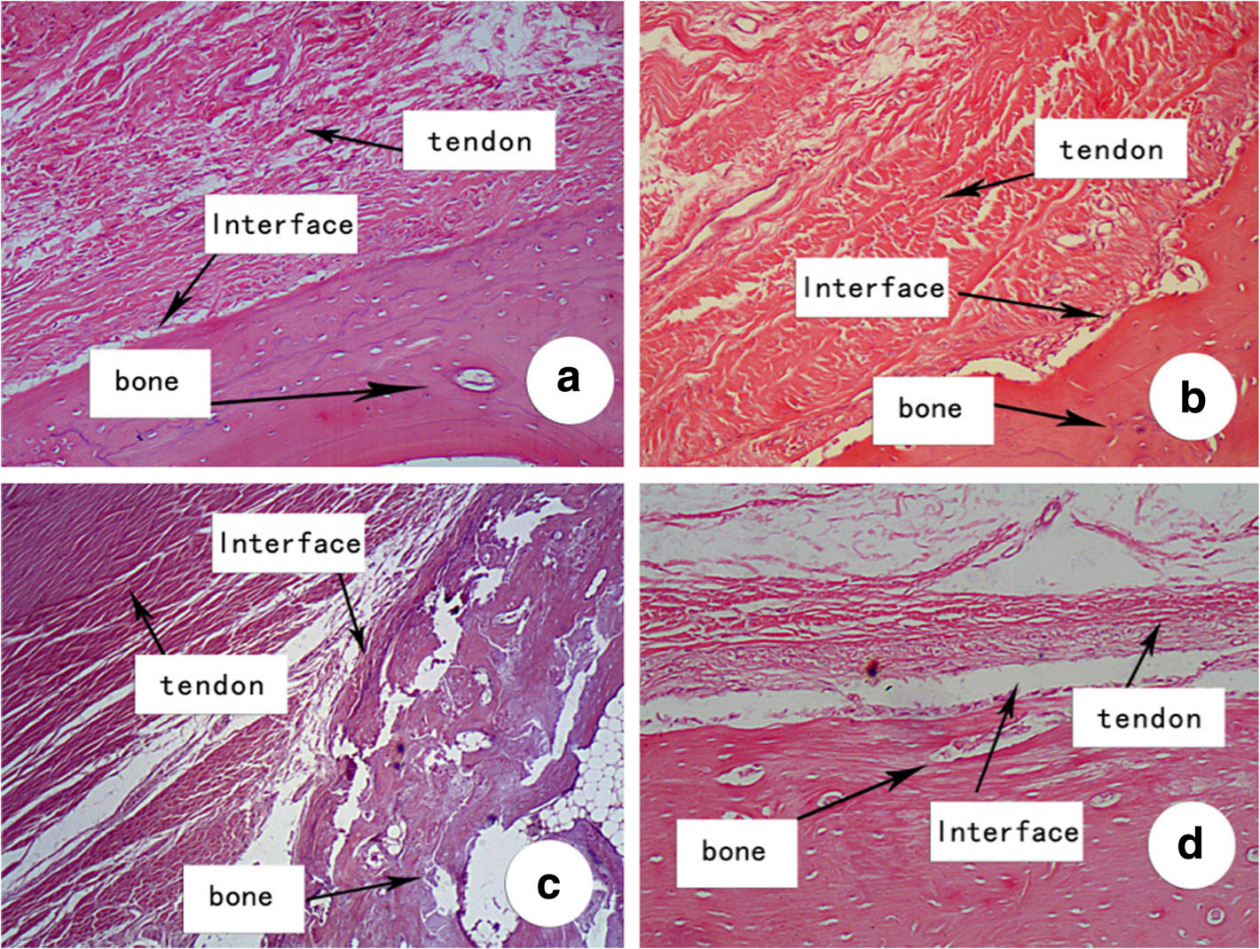
Histological examination of representative week 4 specimen. **a** High aFGF/collagen (4 μg) treatment group. **b** Low aFGF/collagen (1 μg) treatment group. **c** Collagen alone group. **d** Control group. H&E staining, original magnification × 100

Histological sections of the ACL graft tunnel demonstrated differences in histological appearances between grafts that treated with different amount of aFGF/collagen at different time points. At 4 weeks postoperatively, fibrous connective tissue was observed at the tendon–bone interface in all groups. Organized, tightly packed collagen fibers and mature new bone-forming were observed at the tendon–bone interface in the aFGF/collagen treatment groups, especially in the high aFGF concentration group. By comparison, loose and disordered tissues were observed at the tendon–bone interface in the collagen alone and control group with indication of only minimal osteogenesis (Fig. 1).

**Fig. 2:**
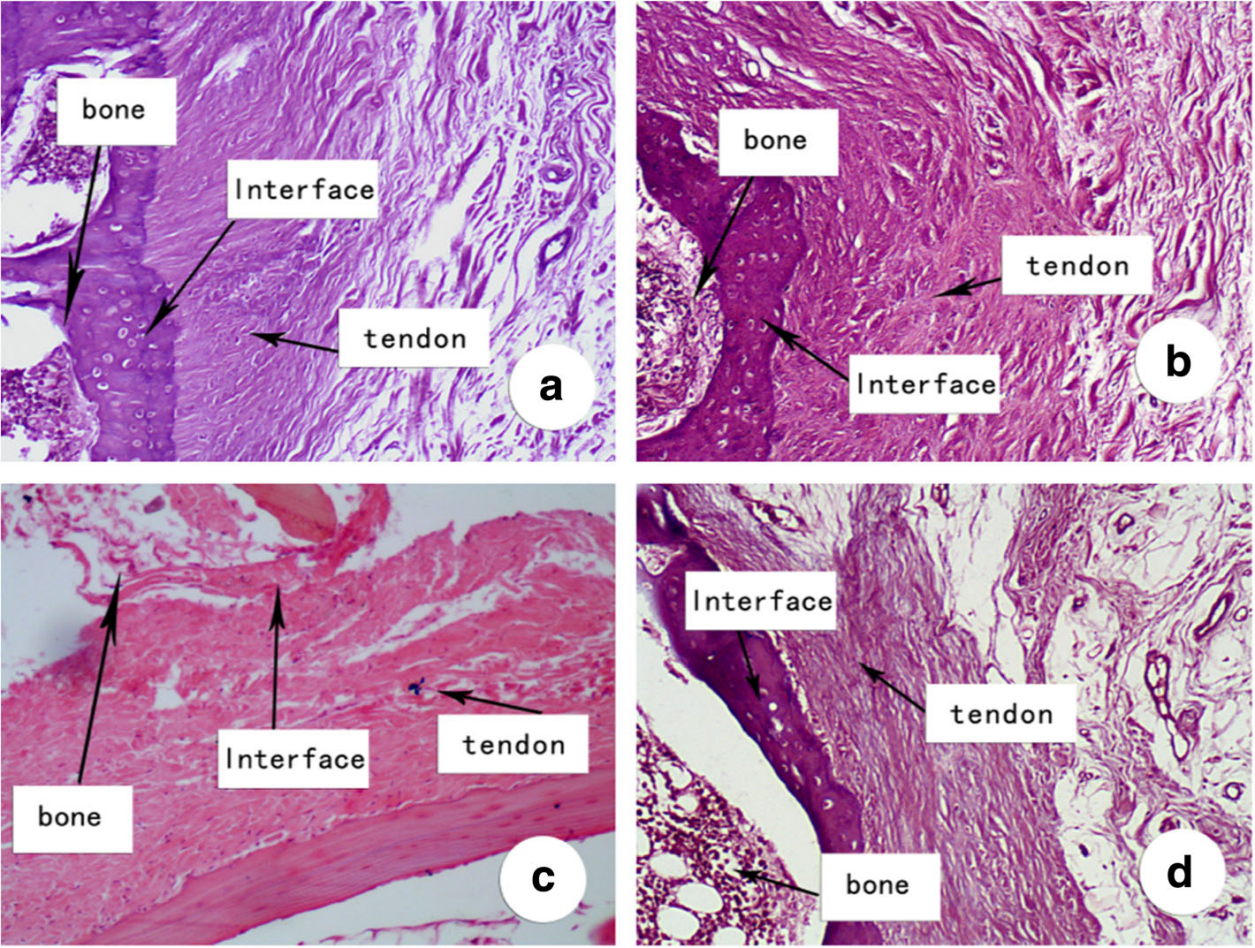
Histological examination of representative week 8 specimen. **a** High aFGF/collagen (4 μg) treatment group. **b** Low aFGF/collagen (1 μg) treatment group. **c** Collagen alone group. **d** Control group. H&E staining, original magnification × 100

In the high aFGF concentration group, densely arranged Sharpey-like fibers were observed vertical to the tendon–bone interface at 8 weeks postoperatively with new bone ingrowth incorporated into the tendon. In the low aFGF concentration group, six out of seven (85.7%) specimens showed Sharpey-like fibers, whereas in the control group, only one out of seven (14.3%) specimens showed Sharpey-like fibers with new bone growth into the tendon and a loose tendon–bone interface (Fig. 2).

**Fig. 3:**
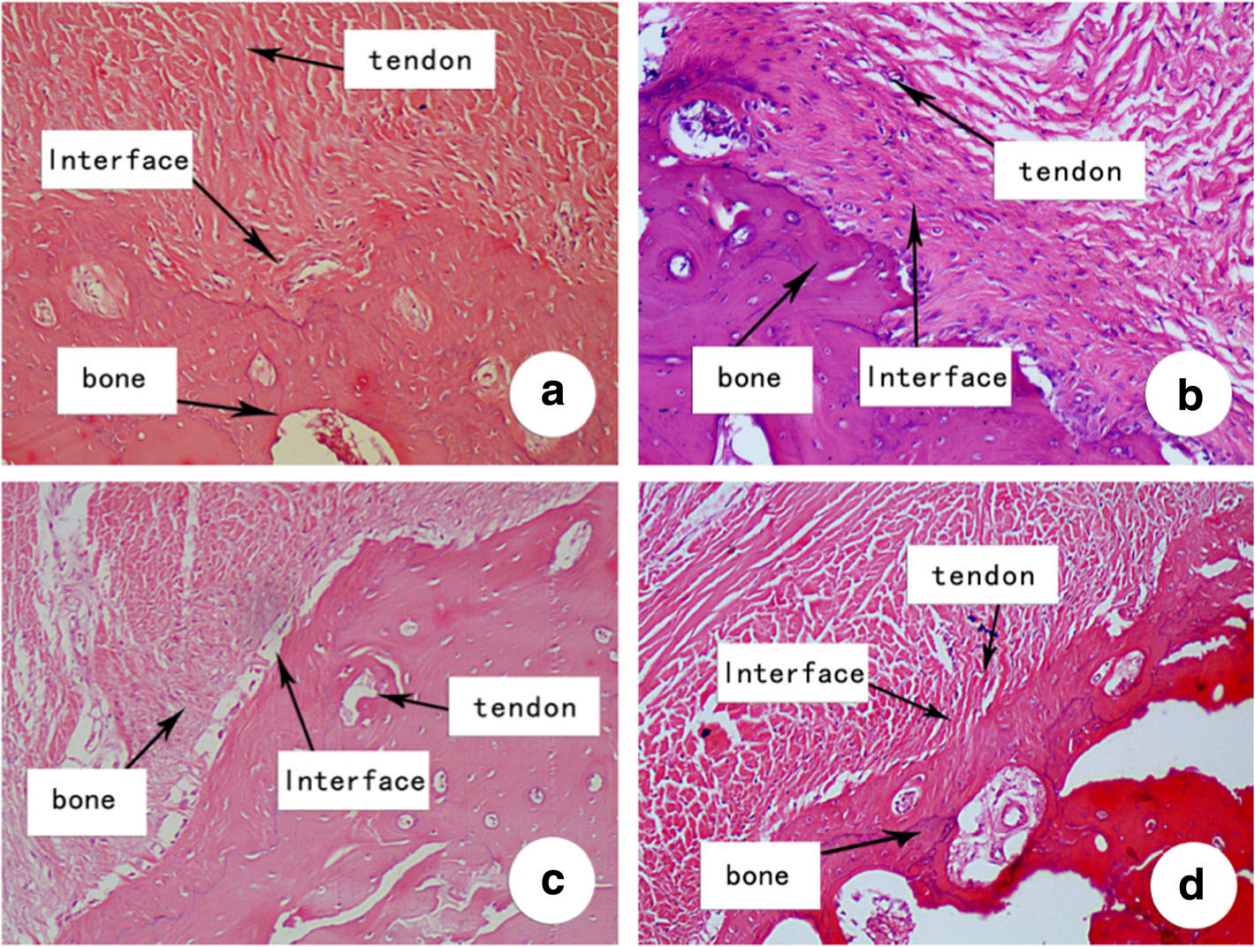
Histological examination of representative week 12 specimen. **a** High aFGF/collagen (4 μg) treatment group. **b** Low aFGF/collagen (1 μg) treatment group. **c** Collagen alone group. **d** Control group. H&E staining, original magnification × 100

At 12 weeks postoperatively, continuous and calcified fibrocartilage had replaced the tendon–bone interface in six out of the seven specimens (85.7%) in the high aFGF concentration group, similar to fibrocartilage enthesis, with fewer Sharpey-like fibers forming a “tide mark”-like structure. In the low aFGF concentration group, a direct enthesis had formed at tendon–bone interface in five of the seven specimens (71.4%); calcified fibrous cartilage was not observed in the other two specimens. The tendon–bone interface gradually matured in the control group, with formation of Sharpey-like fibers and a small amount of poorly differentiated fibrocartilage. No “tide mark” structure was observed in the narrow tendon–bone tissue transition in the collagen alone and control group (Fig. 3). Our quantification of the histological evaluation of all groups also confirmed that the high and low aFGF groups had significantly more tendon–bone interface and “tide mark” structure compared to the control group at both 8 and 12 weeks (Table 1).

**Table 1:** Fisher’s exact test for histological evaluation by H&E staining at all time points

		*n* (%)	Groups (*n*, %)			
			High FGF/collagen	Low FGF/collagen	Collagen	Control
*n*			7	7	7	7
Sharpey-like fiber at 4 weeks	No	3 (10.71)	0 (0)	0 (0)	1 (14.29)	2 (28.57)
	Yes	25 (89.29)	7 (100)	7 (100)	6 (85.71)	5 (71.43)
Fibrocartilage at 8 weeks	No	13 (46.43)	0 (0)	1 (14.29)	6 (85.71)	6 (85.71)
	Yes	15 (53.57)	7 (100)^ab^	6 (85.71)^ab^	1 (14.29)	1 (14.29)
Tide mark at 12 weeks	No	16 (57.14)	1 (14.29)	2 (28.57)	6 (85.71)	7 (100)
	Yes	12 (42.86)	6 (85.71)^ab^	5 (71.43)^a^	1 (14.29)	0 (0)

^a^Compared with the control group at the same time, *P* < 0.05

^b^Compared with the collagen group at the same time point, *P* < 0.05

The percentage of collagen degradation was measured by histological evaluation at all time points (Table 2). As the percentage of collagen degradation increased with time, no significant differences were observed among the different groups at any of the time points. At 4 and 8 weeks, the percentages of collagen degradation were around 25% and 52%, respectively, for all groups. Collagen degradation reached around 76% at 12 weeks, suggesting that approximately 24% of the collagen remained at 12 weeks.

**Table 2:** Percentage of collagen degradation in experimental groups (*n* = 7, average ± SD)

Groups	4 weeks	8 weeks	12 weeks
High aFGF/collagen (4 μg aFGF)	22.71 ± 1.7	52.86 ± 1.07	77.57 ± 0.98
Low aFGF/collagen (1 μg aFGF)	22.86 ± 1.7	52.71 ± 2.06	76.57 ± 2.15
Collagen group	22 ± 1.53	52.29 ± 0.95	75.86 ± 1.35

### Effect of FGF/collagen on ACL graft by biomechanical test

The average load-to-failure, elastic modulus, and stiffness were increased over time significantly in all four groups (*P* < 0.05).

**Fig. 4:**
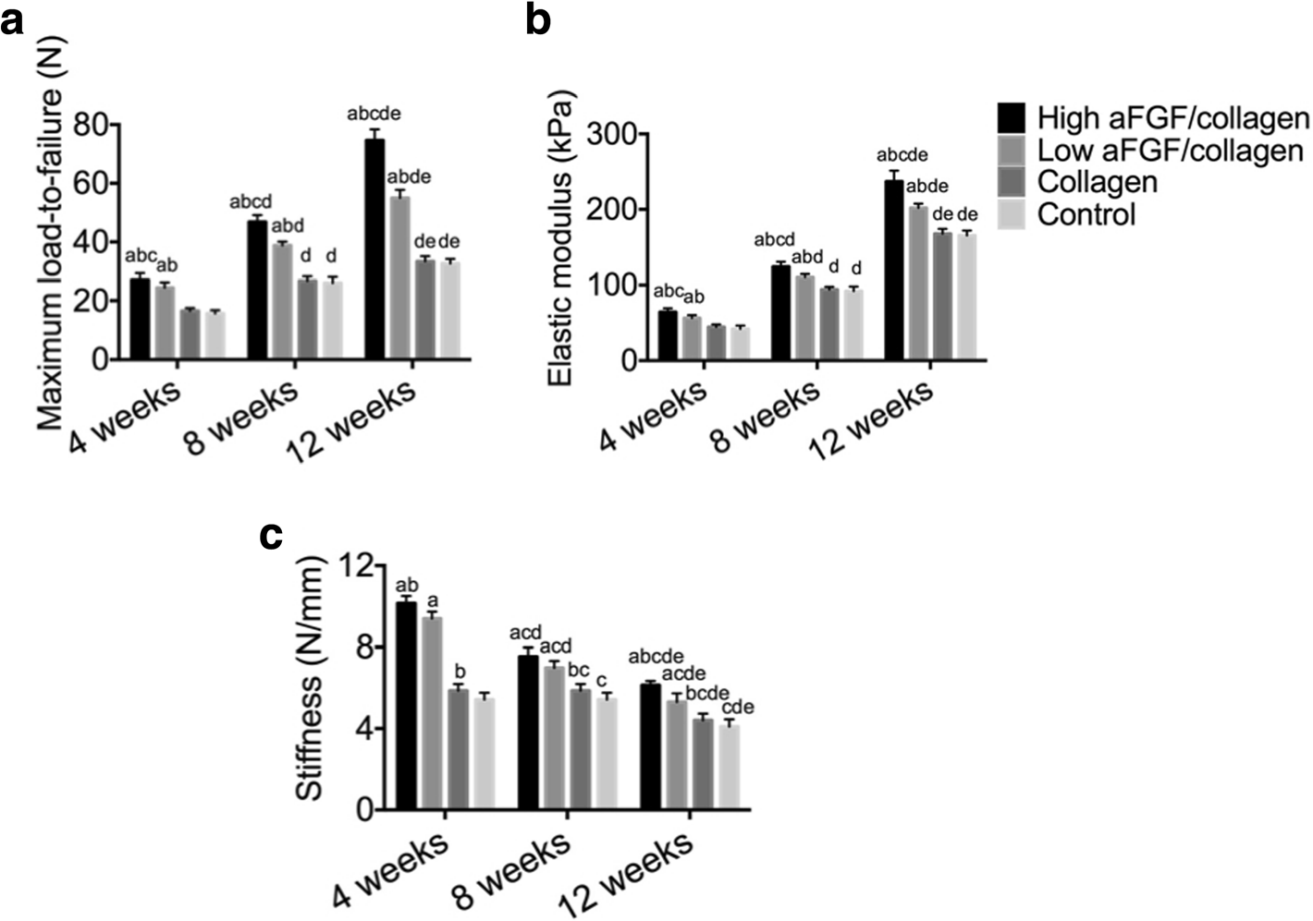
Maximum load-to-failure (**A**), elastic modulus (**B**), and stiffness (**C**) in all groups at different time points compared to a: the control group at the same time, *P* < 0.05; b: the collagen group at the same time point, *P* < 0.05; c: the low aFGF/collagen group at the same time, *P* < 0.05; d: the same group at 4 weeks, *P* < 0.05; and e: the same group at 8 weeks, *P* < 0.05

At 4 weeks, the average load-to-failure, elastic modulus, and stiffness of harvested specimens differed significantly among the four groups. The graft tunnel load-to-failure, elastic modulus, and stiffness were higher in the high and low aFGF/collagen treatment groups compared with the collagen alone and the control group, respectively, with the high aFGF/collagen being the highest. There was no significant difference between the collagen alone and control group (Fig. 4a).

At 8 weeks, the average load-to-failure and elastic modulus in the high aFGF/collagen concentration group remained the highest compared with the other three groups, and the load-to-failure of the low aFGF/collagen concentration group was significantly higher than that of the control group and the collagen alone group, respectively. There was no significant difference between high and low aFGF/collagen concentration group in stiffness (Fig. 4b).

Similarly, at 12 weeks, the high aFGF/collagen concentration group had the highest load-to-failure, elastic modulus, and stiffness. Compared with those of the control group and collagen alone group, the load-to-failure, elastic modulus, and stiffness of the low aFGF concentration group were significantly higher (Fig. 4c).

## Discussion

About 130,000 patients need ACL reconstruction surgery to restore normal structure and function of the knee in the USA each year [13], and 0.7–10% of patients require secondary surgery due to joint instability [14]. Tendon–bone healing has a direct impact on the outcome of ACL reconstruction. In this study, we observed the early beneficial effect of aFGF incorporated within collagen on tendon–bone healing in a rabbit ACL model, especially with the application of aFGF concentration at 0.267 μg/ml.

Histological and biomechanical analyses of tendon–bone healing in a dog model revealed that the healing process occurs by bone ingrowth into the fibrovascular interface tissue that initially forms between the tendon and bone, followed by progressive mineralization of the interface tissue and subsequent bone ingrowth into the outer tendon and incorporation of the tendon graft into the surrounding bone [15]. This healing process involves a variety of biological functions in different cell types. aFGF is a multipotent growth factor that plays an important role in cell proliferation, differentiation, and survival [16]. aFGF was shown to be expressed in fibroblast-like mesenchymal cells and localized to the cambial layer of the periosteum [4]. Increased expression of aFGF and other FGFs was observed in a rabbit model of distraction osteogenesis [8]. Moreover, exogenous delivery of aFGF was shown to promote angiogenesis of adipose-derived mesenchymal stem cells [9] and promote the rabbit ACL cell proliferation in vitro [20].

After ACL reconstruction, the stress load on the tendon during early functional exercise is related to the healing of the tendon–bone interface [18]. The number of Sharpey fibers determines the tensile strength of the transplanted tendon. Consistently, our histological and biomechanical data showed that the aFGF/collagen treatment increased the number of Sharpey fibers, leading to a higher tensile strength compared to that in the control group at 8 weeks after surgery. In addition, fibrocartilage had formed in the treatment groups at 12 weeks postoperatively. The formation of a transitional zone, similar to the normal fibrocartilage connection, suggests that aFGF/collagen treatment can promote healing of the tendon–bone interface early after ACL reconstruction surgery.

Study [23] has suggested that when the diameter of the transplanted tendon is smaller than that of the bone tunnel, the micromotion between the tendons and joint results in synovial fluid flushing in the bone tunnel during joint movement. Compared with direct delivery of exogenous aFGF, collagen carrying aFGF can be used to fill the gap between the tendon and bone, preventing the dramatic decrease in aFGF concentration due to the “synovial fluid flushing” effect. In addition, collagen has osteoinductive and osteoconductive capacities. The incorporation of aFGF within collagen could result in slow release of aFGF for promoting fibrocartilage formation at the tendon–bone interface by decreasing the micromotion between the tendon and bone tunnel and reducing the “synovial fluid effect,” the “wiping effect,” and the “bungee effect” due to the transverse and longitudinal activities of the tendon [10]. Similarly, Gulotta et al. [7] applied a magnesium-based bone adhesive to the bone tunnel and showed that more cartilage and less fibrous tissue were formed at the tendon–bone interface.

## Conclusions

Our data suggested that the application of 4 μg aFGF within collagen as a carrier promoted tendon–bone interface healing in a rabbit ACL reconstruction model. This method of enhancing the healing process may provide an alternative approach to support early rehabilitation of patients and potentially improve the long-term success of ACL reconstruction surgery. Further studies with a larger sample size and characterization of the aFGF release rate are warranted.

## Abbreviation

ACL: Anterior cruciate ligament
